# Supplementary material for: The causal role of circulating inflammatory markers in osteoporosis: a bidirectional Mendelian randomized study
Source: Front Immunol. 2024 Jul 18;15:1412298. doi: 10.3389/fimmu.2024.1412298 (PMC11291241; doi:10.3389/fimmu.2024.1412298)
Supplement: Supplementary file 1 [file DataSheet_1.zip › supplementary material/OSTEOPOROSIS-CIM/GCST90274841.tsv.gz_forest.pdf]

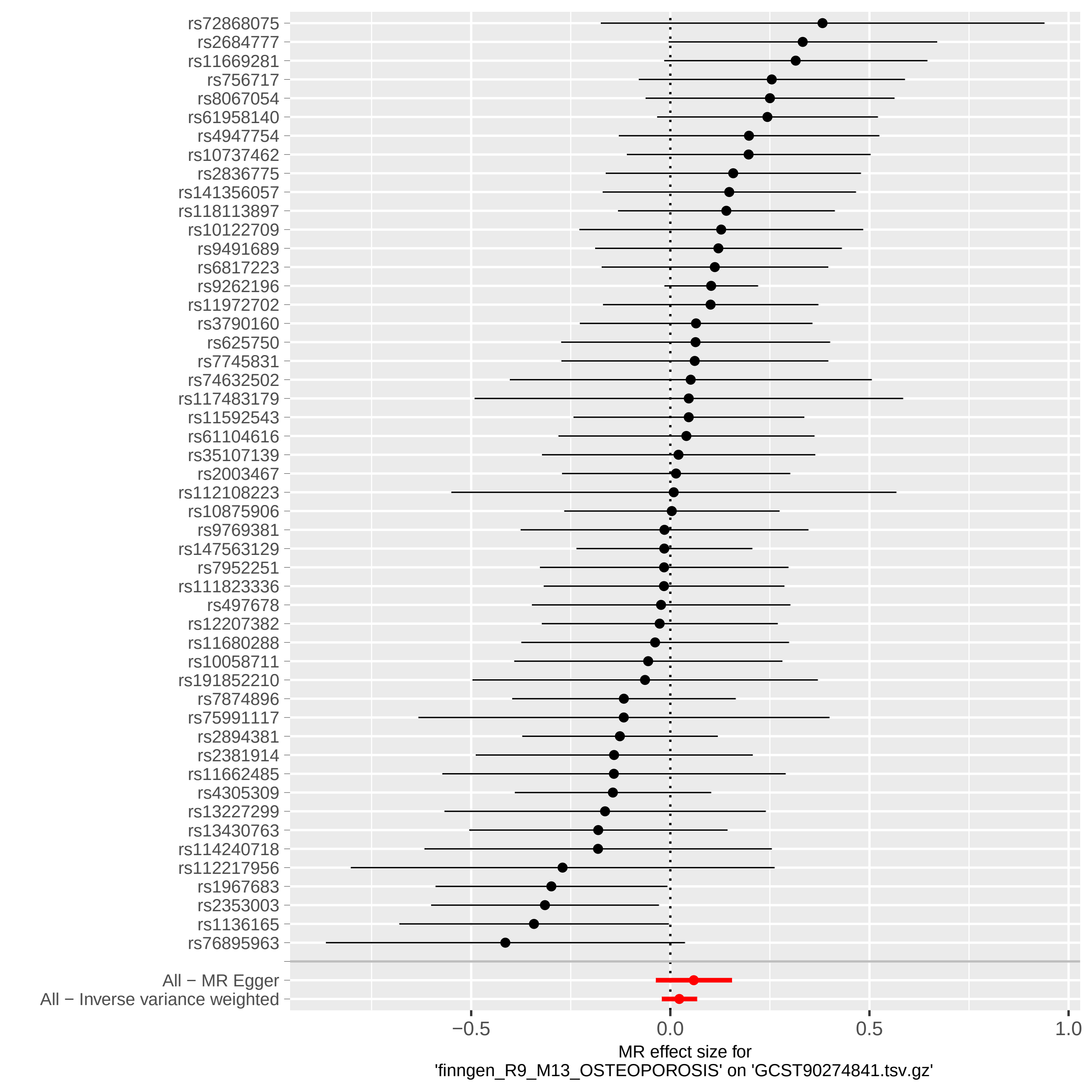
rs72868075
rs2684777
rs11669281
rs756717
rs8067054
rs61958140
rs4947754
rs10737462
rs2836775
rs141356057
rs118113897
rs10122709
rs9491689
rs6817223
rs9262196
rs11972702
rs3790160
rs625750
rs7745831
rs74632502
rs117483179
rs11592543
rs61104616
rs35107139
rs2003467
rs112108223
rs10875906
rs9769381
rs147563129
rs7952251
rs111823336
rs497678
rs12207382
rs11680288
rs10058711
rs191852210
rs7874896
rs75991117
rs2894381
rs2381914
rs11662485
rs4305309
rs13227299
rs13430763
rs114240718
rs112217956
rs1967683
rs2353003
rs1136165
rs76895963
All − MR Egger
All − Inverse variance weighted
−0.5
0.0
0.5
1.0
MR effect size for
'finngen_R9_M13_OSTEOPOROSIS' on 'GCST90274841.tsv.gz'
